# Second-Order Conditioning and Conditioned Inhibition in Different Moments of the Same Training: The Effect of A+ and AX− Trial Number

**DOI:** 10.3389/fnbeh.2021.632548

**Published:** 2021-04-21

**Authors:** Clara Muñiz-Diez, Judit Muñiz-Moreno, Ignacio Loy

**Affiliations:** Department of Psychology, University of Oviedo, Oviedo, Spain

**Keywords:** feature negative discrimination, second-order conditioning, conditioned inhibition, cue interaction, associative learning models

## Abstract

The feature negative discrimination (A+/AX**−**) can result in X gaining excitatory properties (second-order conditioning, SOC) or in X gaining inhibitory properties (conditioned inhibition, CI), a challenging finding for most current associative learning theories. Research on the variables that modulate which of these phenomena would occur is scarce but has clearly identified the trial number as an important variable. In the set of experiments presented here, the effect of trial number was assessed in a magazine training task with rats as a function of both the conditioning sessions and the number of A+ and AX**−** trials per session, holding constant the total number of trials per session. The results indicated that SOC is most likely to be found at the beginning of training when there are many A+ and few AX**−** trials, and CI (as assessed by a retardation test) is most likely to be found at the end of training when there are few A+ and many AX**−** trials. Both phenomena were also found at different moments of training when the number of A+ trials was equal to the number of AX**−** trials. These results cannot be predicted by acquisition-focused associative models but can be predicted by theories that distinguish between learning and performance.

## Introduction

The feature negative discrimination task consists of pairing an initially neutral stimulus (A) both with an unconditioned stimulus (US) and with another initially neutral stimulus (X) in the absence of the US (Pavlov, 1927/1960). This training, represented as A+/AX**−**, can result either in X gaining excitatory properties, a phenomenon known as second-order conditioning (SOC), or in X acquiring inhibitory properties, a phenomenon known as conditioned inhibition (CI). The fact that opposite results can be obtained constitutes a challenge for current associative learning theories, as most of them were developed in the light of cue competition phenomena. For instance, the highly influential Rescorla and Wagner (1972) model was aimed to account for learning phenomena such as blocking (Kamin, 1968) or degraded contingency (Rescorla, 1968). It is for this reason that these theories readily explain the phenomenon of CI, which can be considered a form of cue competition. However, cue facilitation effects, such as SOC, are not predicted by most of these models. An exception to this would be the models that are able to differentiate between acquisition and performance, such as the one proposed by Stout and Miller (2007) or Pineño (2007). Thus, research on the circumstances under which cue competition or cue facilitation emerge is of great theoretical importance (for a review, see Urcelay, 2017).

The circumstances that lead to SOC or CI are still unclear, but previous research has focused in three main variables: the temporal relationship of the stimuli in the compound (AX**−**), the number of trials employed, and the order of presentation of the AX**−** trials in relation to the A+ trials. Regarding the temporal relationship of the stimuli in the compound, Pavlov (1927/1960) pointed out some methodological details that need to be taken into account to produce one phenomenon or the other. For example, if the stimuli in the compound (AX) overlap to any extent, CI is observed, but if the new stimulus (X; hereafter referred to as second-order stimulus) is presented just before the onset of the conditioned stimulus (CS; A; hereafter referred to as first order stimulus), then CI is observed less frequently. On the contrary, if the interval between them is increased, SOC is found. Pavlov also mentioned that the duration of the interval should be increased according to the increasing intensity of the second-order stimulus in order to achieve SOC. However, this statement was proved wrong in subsequent studies with different procedures in which SOC was found in spite of overlapping presentations of the first- and second-order stimuli (e.g., Maisiak and Frey, 1977; Rescorla, 1982). Kehoe et al. (1981), in a study of the rabbits’ nictitating membrane response, found an inverse relationship between responding to the second-order stimulus and the interval between the first- and second-order stimuli. This result was replicated by Gibbs et al. (1991) with the same procedure. Taken together, these conflicting results suggest that the temporal relationship between the stimuli in the compound is not a critical variable to find either SOC or CI and that its effect, if any, is modulated by other experimental parameters.

As already mentioned, another variable that has been examined is the number of trials. Kehoe et al. (1981) also found that the excitatory response to X followed an inverted U shape as the sessions progressed, although the response was significantly higher than in the control group throughout the experiment, which indicated that SOC was maintained. Gibbs et al. (1991), again using the rabbits’ nictitating membrane response, assessed this issue varying the number of AX**−** trials per session (5, 15, 25, or 50 for each group, respectively), while the number of A+ trials was kept fixed (30). The results indicated that SOC occurred in all groups, as confirmed by the significant differences with the respective unpaired control groups, and that responding followed an inverted U shape as a function of the number of trials (i.e., responding was greater in groups that received 15 and 25 AX**−** trials per session). Despite this, none of these studies clearly demonstrated CI, as tests for the inhibitory properties were not performed. Rescorla (1972, 1973) and Holland and Rescorla (1975), employing, respectively, a conditioned suppression and a magazine training procedure in rats, showed excitatory conditioning to X at the beginning of training that decreased as the sessions progressed. After training, the inhibitory properties of X were confirmed by a summation test, i.e., the stimulus was able to reduce responding to an excitatory stimulus (transfer excitor) that had been trained independently. All in all, the studies that assess the effect of number of trials indicate that SOC is found early in training and tends to fade out after a certain number of sessions, eventually turning into CI.

Finally, the third variable that interacts with the effect of the number of trials is whether training is performed in two phases or not (Yin et al., 1994). SOC is usually found when A is reinforced in a phase previous to AX**−** training (e.g., Rizley and Rescorla, 1972), whereas CI is usually found when A+ and AX**−** trials are interspersed in one single phase (e.g., Pavlov, 1927/1960). Yin et al. (1994) carried out three experiments to determine if the number of trials and the use of phases were of significant importance for the finding of SOC or CI. The results of these experiments indicated that SOC was found only with few AX**−** trials (a total of four trials across training), no matter if they were presented after or interspersed with 96 A+ trials, and that CI is found when there are many AX**−** trials (48 across training) interspersed with 96 A+ trials. Stout et al. (2004), using the same procedure, examined the effect of the temporal relationship of A and X in those trials. They presented, across training, either few (four), intermediate (20), or many (100) AX**−** trials interspersed with 48 A+ trials, and in the AX**−** trials, the stimuli were presented either serially (the offset of X coincided with the onset of A) or simultaneously (X and A overlapped). The results indicated that the two variables interacted significantly, so with a few trials both temporal arrangements led to SOC, with many trials both temporal arrangements led to CI, and with an intermediate number of trials, if the AX**−** compound was presented serially, it led to SOC, and if it was presented simultaneously, it led to CI.

The number of trials is a variable that has been studied in two different ways in the reviewed studies. Some of the studies took into account the number of sessions and found that the development of SOC is attenuated with extended training and that, at the end of training, CI is developed. On the other hand, some other studies manipulated the number of AX**−** trials per session. Importantly, in this case, the total number of trials and the intertrial interval (ITI) differed between groups, thus being potentially confounding variables. The present set of experiments aimed to further examine the transition from SOC to CI throughout the sessions by holding constant the total number of trials per session and ITI and by manipulating the number of A+ and AX**−** trials per session. Whereas the literature clearly shows that the number of trials is a key variable in finding SOC or CI, the effect of the temporal relationship of the stimuli in the compound and the order of presentation of AX**−** trials in relation to A+ trials is not so clear. These variables were out of the scope of the present experiments, so A+ trials were presented interspersed with AX**−** trials as in the study by Stout et al. (2004), and the AX**−** compound was presented in a simultaneous way as in the study by Yin et al. (1994).

## Experiment 1

The design of Experiments 1–3 is depicted in Table 1. In Experiment 1, two groups of rats were trained in a magazine procedure, where the US was a food pellet, and the conditioned response (CR) was the number of entries into the food delivery site in the presence of the CS. During training, both groups received 14 A+ trials and two non-reinforced compound trials per session across 20 sessions. In each session, X**−** alone trials were included to test the CR controlled by this stimulus. The difference between groups was that, in one group, the compound was formed by A and X, whereas in the other group, the compound was formed by B and X, thus acting as a control for SOC and CI. After conditioning, both groups were tested for inhibitory properties using a retardation test, i.e., presenting X followed by the US. It was expected that the subjects in the experimental group would develop a higher responding to X in the first sessions of the experiment, which would indicate SOC, and that, with extended training, responding would equate with the control group. Regarding CI, according to the results reported by Rescorla (1972, 1973) and by Holland and Rescorla (1975), it would be expected to occur, but based on the results by Yin et al. (1994) and Stout et al. (2004), with few AX**−** trials only SOC would be expected.

**TABLE 1:** Experimental designs.

**Experiment**	**Group**		**Conditioning**	**Retardation test**
EXP1	14-2	Experimental	14A+ / 12F− / 2AX− / 2X−	10X+
		Control	14A+ / 12F− / 2BX− / 2X−	10X+
Exp2	8-8	Experimental	8A+ / 6F+ / 6F− / 8AX− / 2X−	10X+
		Control	8A+ / 6F+ / 6F− / 8BX− / 2X−	10X+
Exp3	5-11	Experimental	5A+ / 9F+ / 3F− / 11AX− / 2X−	10X+
		Control	5A+ / 9F+ / 3F− / 11BX− / 2X−	10X+

*A represents tone presentation, B represents light presentation, AX represents tone-click compound presentation, F represents lever presentation and X represents click presentation. The numbers before the letters indicate the number of trials that the stimulus was presented in each session. The + symbol represents that the stimuli were followed by a food pellet and the − symbol represents that the stimuli were not followed by a food pellet.*

### Method

#### Subjects

The sample size needed was first calculated using G^∗^Power (Faul et al., 2007). The total sample size needed to achieve an effect size *f* of 0.25, with the level of significance α = 0.05 and power *1*-β = 0.95, was 14. Two subjects were added in case there was some sample loss (which was not the case for this experiment), so the subjects were 16 experimentally naive male Wistar rats that were 100 days old and had an *ad libitum* weight of 408 g (range, 343–474 g). All procedures related to the maintenance and use of animals were in accordance with the European Law of Animal Welfare and were approved by the Animal Welfare Committee of the University of Oviedo. They were housed in cages, each of which contained four rats that received the same training during the experiment. The weight of the animals was gradually reduced by controlled feeding to 85% of their individual free-feeding weights and was kept at that level throughout the experiment. Each day, in the housing room, there was 12 h of light, beginning at 8 a.m. The experiment was run during this light phase.

#### Apparatus

Eight identical conditioning chambers (24 × 29 × 38 cm: height × width × depth; Med Associates) were placed in a sound- and light-attenuating shell that incorporated a ventilation fan, which maintained the background noise at 62 dB(A). Background light was turned off for the experiment. The front and back walls were constructed from aluminum, the side walls and the ceiling were of clear methacrylate, and the floor was formed from 0.4 cm stainless steel rods, spaced 1 cm apart. A recessed food well (6 × 3.5 × 6 cm) was placed at the center of the front wall, 0.5 cm above the floor. Foods pellets (45 mg, Test Diet-MLab Rodent Tablet) were delivered to the food well and played the role of the US. The food well was equipped with photocells that allowed the presence of the rat in the well to be automatically recorded, playing the role of the response. A speaker that produced a 600 Hz and 76 dB(A) tone was mounted on the front wall, 8 cm over the food magazine. Above this speaker, there was another speaker that generated a second auditory stimulus: a 3,000 Hz and 82-dB(A) intermittent click. A 2 W and 24 V light was situated just above the food magazine. A stainless steel retractable lever (4.8 × 0.55 × 1.9 cm) was located 3 cm to the left of the food well. The depression of the lever was not recorded as a response nor had any scheduled consequence. The presence of the lever in the chamber was used as a stimulus, and when not active, it was retracted into the chamber wall. The tone, click, light, and presence of the lever all lasted 10 s and were used as stimuli as described in the procedure section below.

#### Procedure

Rats were randomly assigned to two groups of eight subjects each and then received 4 days of magazine training followed by 20 sessions of conditioning followed by four sessions of retardation test. The groups were labeled 14-2Exp and 14-2Ctrl.

##### Magazine training

On days 1–4, the subjects received a 20 min session of magazine training. In each session, food pellets were delivered according to a variable time 120 s schedule. Four pellets were placed in the magazine before the beginning of these sessions.

##### Conditioning

Conditioning began on day 5 and continued throughout day 24 (a total of 20 sessions). Each session lasted 52 min. The subjects in group 14-2Exp received 14 tones followed by a food pellet (A+), two non-reinforced tone-click compounds (AX**−**), 12 non-reinforced presentations of the lever (F**−**), and two non-reinforced clicks (X**−**) per session. Stimuli were presented in random order within the session. The ITI had a mean duration of 80 s (range, 50–110 s). The first and last 100 s had no event scheduled. Training for 14-2Ctrl group was identical to 14-2Exp, except that two light-click compounds (BX**−**) were presented instead of two tone-click compounds (AX**−**). The function of the lever presentations was twofold: they were included to control the total amount of reinforcement received per session across the experiments presented here, in such a way that all subjects received 14 food pellets per session in all experiments, and they also allowed to slow down the development of excitatory responding to A. This, as shown in preliminary unpublished studies from our laboratory, was necessary to observe excitatory responding to X. Click-alone presentations were included to test the CR to this stimulus.

##### Retardation Test

On days 25–28, all subjects received a 20-min retardation test. In each session, 10 clicks followed by a food pellet (X+) were presented, with a mean ITI of 80 s (range, 50–110 s).

#### Data Analysis

Food well entries were registered during the 10 s that preceded the presentation of the CS and during the presentation of the CS itself. The CR controlled by the CS was computed as the difference in responding during the CS and the pre-CS periods, which was averaged for each session. The rationale for choosing this measure was that it allows to control for the general activity differences that can be seen between subjects. All the analyses reported here were performed on the mean differences per session. SPSS 24 (IBM Corp., 2016) was used to analyze the data. The analyses were mixed-model ANOVAs. The level of significance used was α = 0.05. The effect sizes for ANOVAs are reported as partial Eta-square ($\eta_{p}^{2}$).

### Results

**FIGURE 1:**
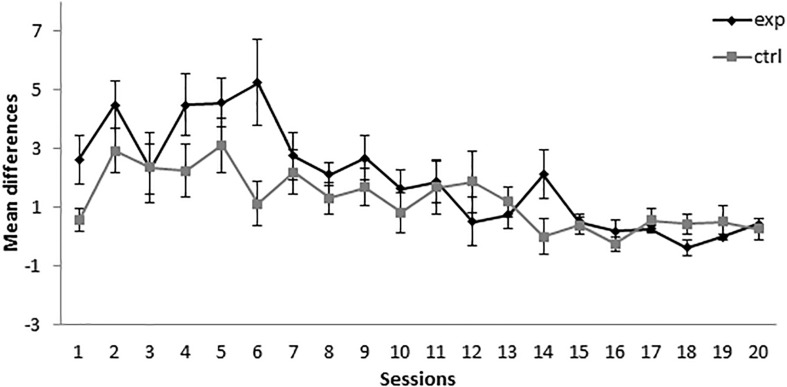
Conditioning phase in Experiment 1. PreX-X differences (±SEM), averaged for the two X**−** presentations per session in conditioning, are displayed. The black line represents the group that was trained with 14 A+, two AX**−**, 12 F**−**, and two X**−** presentations per session in conditioning. The gray line represents the group that was trained with 14 A+, two BX, 12 F**−**, and two X**−** presentations per session.

As can be seen in Figure 1, during the first sessions of the conditioning phase, the subjects in group 14-2Exp, the one in which A+ was presented 14 times and AX**−** was presented twice, showed higher responding to X than the control group, for which BX**−** instead of AX was used as a compound. Responding in group 14-2Exp matched the responding in group 14-2Ctrl at around session 7. A mixed-model ANOVA with a between-subjects factor Group (experimental or control) and a within-subjects factor Session found a significant main effect of Session, *F*(19,266) = 7.001, *p* < 0.001, $\eta_{p}^{2}$ = 0.333, and of Session × Group interaction, *F*(19,266) = 1.876, *p* = 0.016, $\eta_{p}^{2}$ = 0.118, but not a main effect of Group, *F*(1,14) = 3.175, *p* = 0.096, $\eta_{p}^{2}$ = 0.185. Bonferroni-corrected pairwise comparisons for the interaction showed that there were significant differences between the experimental and control groups in session 1, *MD* = 2.063, *SE* = 0.912, *p* = 0.04, and session 6, *MD* = 4.125, *SE* = 1.663, *p* = 0.026. These analyses indicate that the subjects in the group that received 14 A+ and two AX**−** presentations per session developed a significantly higher response to the click (X) than the subjects that received 14 A+ and two BX**−** presentations per session in sessions 1 and 6, a result that is congruent with the development of SOC.

**FIGURE 2:**
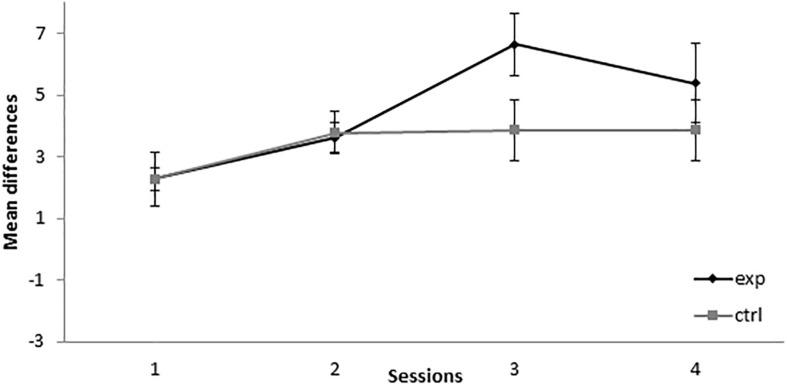
Retardation test in Experiment 1. PreX-X differences (±SEM), averaged for the 10 X+ presentations per session in the retardation test, are displayed. The black line represents the group that was trained with 14 A+, two AX**−**, 12 F**−**, and two X**−** presentations per session in conditioning. The gray line represents the group that was trained with 14 A+, two BX, 12 F**−**, and two X**−** presentations per session. In the retardation test, both groups received 10 X+ presentations per session.

In the retardation test, responses to X in the group that received the 14A+/2AX**−** treatment (group 14-2Exp) showed no differences with the control group in the first two sessions. In contrast, responding to X by group 14-2Exp was higher than in the control group in sessions 3 and 4, as can be seen in Figure 2. A mixed-model ANOVA with the between-subjects factor Group (experimental or control) and the within-subjects factor Session found a significant main effect of Session, *F*(3,42) = 7.344, *p* < 0.001, $\eta_{p}^{2}$ = 0.344, but not of Group, *F*(1,14) = 1.2, *p* = 0.292, $\eta_{p}^{2}$ = 0.079, or Session × Group interaction, *F*(3,42) = 2.084, *p* = 0.117, $\eta_{p}^{2}$ = 0.13. This analysis indicated that both groups increased their responding to X over sessions in a similar way. The absence of a significant group effect in the analysis indicated that, in group 14-2Exp, X did not gain inhibitory properties.

Taken together, the results of this experiment indicated that the group that was trained with 14 A+ and two AX**−** presentations per session showed an increase in responding to X in sessions 1 and 6, which might indicate the development of SOC in those sessions. However, the absence of a difference between the two groups in the retardation test indicates that it did not develop CI. These results are consistent with the previous literature (Rescorla, 1972, 1973; Holland and Rescorla, 1975), as excitatory responding to X is developed in two sessions at the beginning of training and disappears with extended training. The absence of CI is not consistent with the results obtained by Rescorla (1972, 1973) and Holland and Rescorla (1975) but is consistent with the results found by Yin et al. (1994) and Stout et al. (2004) when they used few AX**−** trials.

## Experiment 2

Experiment 2 aimed to find excitatory properties at the beginning of training and inhibitory properties at the end. In order to achieve this, the number of A+ trials was lowered from 14 to eight, and the number of AX**−** trials was increased from two to eight, thus maintaining the total number of trials presented per session equal to the total number of trials per session presented in the previous experiment. In short, the experimental group received eight A+ and eight AX**−** trials, whereas the control group received eight A+ and eight BX**−** trials. This experiment included a retardation test identical to the ones employed in the experiments above.

### Method

#### Subjects and Apparatus

The sample size was calculated as in the previous experiment, but in this case, one of the rats died. Hence, the subjects were 15 experimentally naive male Wistar rats that were 105 days old and that had an *ad libitum* weight of 459 g (range, 420–515 g). Housing, deprivation schedule, and apparatus were identical to those of Experiment 1.

#### Procedure

The rats were randomly assigned to two groups and then received four days of magazine training followed by 20 sessions of conditioning and four sessions of retardation test. The groups were labeled 8-8Exp and 8-8Ctrl. Group 8-8Exp had eight subjects and group 8-8Ctrl had seven subjects.

The subjects in group 8-8Exp received eight tones followed by a food pellet (A+), eight non-reinforced tone-click compounds (AX**−**), six non-reinforced presentations of the lever (F**−**), six presentations of the lever followed by a food pellet (F+), and two non-reinforced clicks (X**−**) per session. Six of the 12 lever presentations were reinforced in order to equate the number of reinforcers received per session with that of the previous experiment. The training for 8-8Ctrl group was identical to the one for 8-8Exp, except that eight light-click compounds (BX**−**) were presented instead of eight tone-click compounds (AX**−**). All other details were identical to those of Experiment 1.

### Results

**FIGURE 3:**
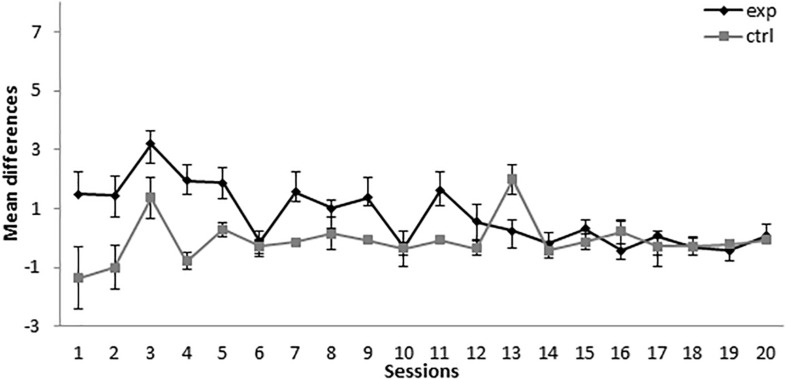
Conditioning phase in Experiment 2. PreX-X differences (±SEM), averaged for the two X**−** presentations per session in conditioning, are displayed. The black line represents the group that was trained with eight A+, eight AX**−**, six F**−**, six F+, and two X**−** presentations per session in conditioning. The gray line represents the group that was trained with eight A+, eight BX**−**, six F**−**, six F+, and two X**−** presentations per session.

During the first 11 sessions of conditioning, the subjects in group 8-8Exp (i.e., trained with 8A+ and 8AX**−**) showed a higher responding to X than the subjects in the control group (8-8Ctrl), for which BX**−** instead of AX**−** was used as a compound (see Figure 3). A mixed-model ANOVA found statistically significant differences for the main effects Session, *F*(19,247) = 3.777, *p* < 0.001, $\eta_{p}^{2}$ = 0.225, Group, *F*(1,13) = 18.485, *p* = 0.001, $\eta_{p}^{2}$ = 0.587, and Session × Group interaction, *F*(19,247) = 3.081, *p* < 0.001, $\eta_{p}^{2}$ = 0.192. Bonferroni-corrected pairwise comparisons for the interaction showed that there were significant differences between the experimental and the control groups in session 1, *MD* = 2.857, *SE* = 1.258, *p* = 0.041, session 2, *MD* = 2.438, *SE* = 0.992, *p* = 0.029, session 3, *MD* = 1.83, *SE* = 0.79, *p* = 0.038, session 4, *MD* = 2.723, *SE* = 0.652, *p* = 0.001, session 5, *MD* = 1.589, *SE* = 0.606, *p* = 0.021, session 7, *MD* = 1.705, *SE* = 0.712, *p* = 0.032, session 11, *MD* = 1.696, *SE* = 0.682, *p* = 0.027, and session 13, *MD* = −1.75, *SE* = 0.6, *p* = 0.012. The experimental group showed an increase in responding to X in sessions 1, 2, 3, 4, 5, 7, and 11, which is congruent with subjects acquiring SOC. There was also a significant higher responding in control group in session 13.

**FIGURE 4:**
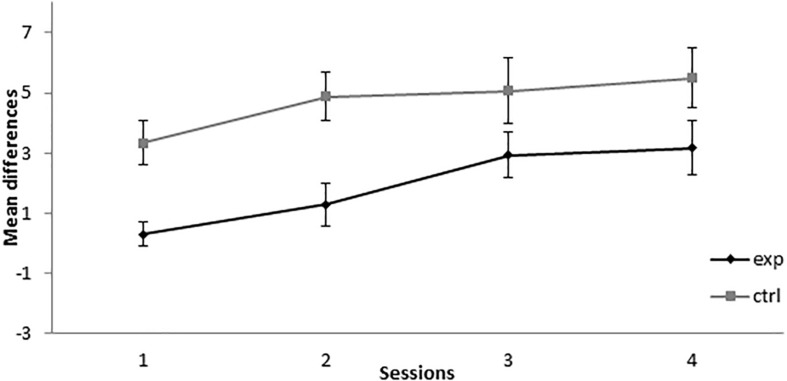
Retardation test in Experiment 2. PreX-X differences (±SEM), averaged for the 10 X+ presentations per session in retardation, are displayed. The black line represents the group that was trained with eight A+, eight AX**−**, six F**−**, six F+, and two X**−** presentations per session in conditioning. The gray line represents the group that was trained with eight A+, eight BX**−**, six F**−**, six F+, and two X**−** presentations per session. In the retardation test, both groups received 10 X+ presentations per session.

As can be seen in Figure 4, during the retardation test, the experimental group showed a lower responding than the control group across all sessions. A mixed-model ANOVA found statistically significant effects for the main effects Session, *F*(3,39) = 10.688, *p* < 0.001, $\eta_{p}^{2}$ = 0.451, and Group, *F*(1,13) = 7.745, *p* = 0.016, $\eta_{p}^{2}$ = 0.373, but not for the Session × Group interaction, *F*(3,39) = 0.931, *p* = 0.435, $\eta_{p}^{2}$ = 0.067. This analysis showed that both groups increased their responding to X across sessions, but there was retardation in the acquisition of conditioning in the experimental group compared with the control group, thus indicating that X gained inhibitory properties.

The results of this experiment altogether indicated that, when 8A+ and 8AX**−** trials are presented per sessions, responding to X increases in the first sessions, a result consistent with SOC, and that, at the end of training, X showed a significant retardation in conditioning when paired with the US, thus indicating inhibitory properties consistent with CI. These results are consistent with the results found by Rescorla (1972, 1973) and Holland and Rescorla (1975), as SOC was found at the beginning of training, fading as sessions progressed, and CI was found at the end of training. The aforementioned authors demonstrated CI based on a summation test, whereas in this experiment CI was demonstrated based on a retardation test.

## Experiment 3

Taking into account that Yin et al. (1994) and Stout et al. (2004) found that, with many trials, only CI was developed, it would be interesting to assess if a greater number of AX**−** trials would prevent that development of SOC while not affecting the development of CI. Experiment 3 was designed to assess this question by increasing the number of AX**−** trials and decreasing, accordingly, the number of A+ trials. In order to achieve this, the experimental group of this experiment received five A+ and 11 AX**−** presentations per session. It was compared with a control group that received five A+ and 11 BX**−** presentations per session.

### Method

#### Subjects and Apparatus

The sample size was calculated as in the previous experiments. However, two rats died, so the subjects were 14 experimentally naive male Wistar rats that were 71 days old and had an *ad libitum* weight of 247 g (range, 224–279 g). Housing, deprivation schedule, and apparatus were identical to those of experiments 1 and 2.

#### Procedure

The rats were randomly assigned to two groups of seven subjects each and then received four days of magazine training followed by 20 sessions of conditioning and four sessions of retardation test. The groups were labeled 5-11Exp and 5-11Ctrl.

The subjects in group 5-11Exp received five tones followed by a food pellet (A+), 11 non-reinforced tone-click compounds (AX**−**), three non-reinforced presentations of the levers (F**−**), nine presentations of the lever followed by a food pellet (F+), and two non-reinforced clicks (X**−**) per session. The training for the 5-11Ctrl group was identical to the one of 5-11Exp, except that 11 light-click compounds (BX**−**) were presented instead of 11 tone-click compounds (AX**−**). All other details were identical to experiments 1 and 2.

### Results

**FIGURE 5:**
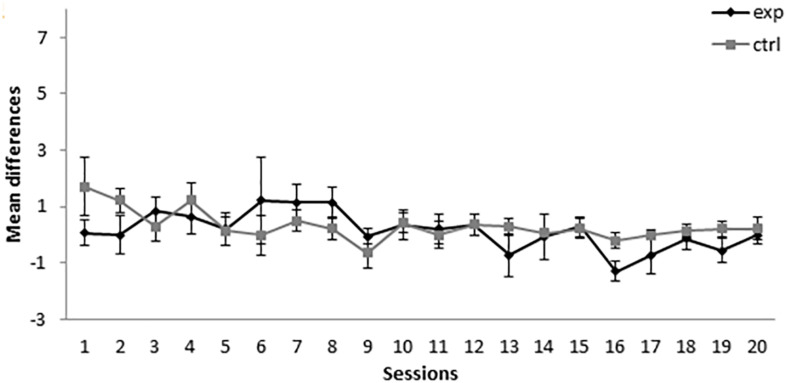
Conditioning phase in Experiment 3. PreX-X differences (±SEM), averaged for the two X**−** presentations per session in conditioning, are displayed. The black line represents the group that was trained with five A+, 11 AX**−**, three F**−**, nine F+, and two X**−** presentations per session in conditioning. The gray line represents the group that was trained with five A+, 11 BX**−**, three F**−**, nine F+, and two X**−** presentations per session.

As can be seen in Figure 5, the subjects in groups 5-11Exp (for which A+ was presented five times and AX**−** was presented 11 times) and 5-11Ctrl (for which BX**−** was used as a compound instead of AX**−**) showed a similar level of responding throughout the conditioning phase of the experiment. A mixed-model ANOVA with the between-subjects factor Group (experimental or control) and the within-subjects factor Session found no statistically significant differences nor a significant interaction [Session: *F*(19,228) = 1.420, *p* = 0.119, $\eta_{p}^{2}$ = 0.106, Group: *F*(1,12) = 0.681, *p* = 0.425, $\eta_{p}^{2}$ = 0.054, Session × Group: *F*(19,228) = 0.941, *p* = 0.533, $\eta_{p}^{2}$ = 0.073]. The absence of significant differences indicates that X did not acquire excitatory properties at any point of the experiment.

**FIGURE 6:**
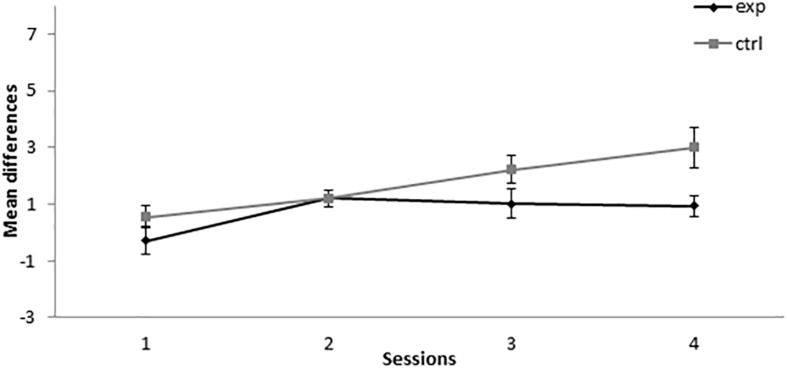
Retardation test in Experiment 3. PreX-X differences (±SEM), averaged for the 10 X+ presentations per session in retardation, are displayed. The black line represents the group that was trained with five A+, 11 AX**−**, three F**−**, nine F+, and two X**−** presentations per session in conditioning. The gray line represents the group that was trained with eight A+, five A+, 11 BX**−**, three F**−**, nine F+, and two X**−** presentations per session. In the retardation test, both groups received 10 X+ presentations per session.

In the retardation test, 5-11Exp showed a lower level of responding than the control group in all sessions except for session 2, as can be seen in Figure 6. A mixed-model ANOVA with the between-subjects factor Group (experimental or control) and the within-subjects factor Session found a significant main effect of Session, *F*(3,36) = 7.18, *p* = 0.001, $\eta_{p}^{2}$ = 0.374, and of Group, *F*(1,12) = 5.692, *p* = 0.034, $\eta_{p}^{2}$ = 0.322, but not of the Session × Group interaction, *F*(3,36) = 2.091, *p* = 0.119, $\eta_{p}^{2}$ = 0.148. This analysis indicates that, even when both groups increased their responding to X across sessions, there is a consistently lower responding in the group that was trained with five A+ and 11 AX**−** trials, thus indicating that CI was developed in the 5-11Exp group.

All in all, the results of Experiment 3 showed that the group that was trained with five A+ and 11 AX**−** presentations per session did not developed SOC at any point of the experiment. However, in the retardation test, the pattern of the results was congruent with the development of CI in group 5-11Exp. These results are consistent with the results reported by Yin et al. (1994) and by Stout et al. (2004) in the experiments where many AX**−** trials were used as they did find CI but not SOC.

## General Discussion

These experiments show that, when 14 A+ trials and two AX**−** trials were presented in each of the training sessions (Experiment 1), the subjects showed an increase in responding to X congruent with SOC in sessions 1 and 6 that faded out in the last sessions. Moreover, these subjects did not show retardation of conditioning to X at the end of training, thus indicating that CI was not developed. Contrastingly, the subjects that were trained with eight A+ and eight AX**−** trials in each session (Experiment 2) showed an increase in responding to X in the first half of the training sessions, consistent with a SOC effect, and a retardation of conditioning to X in the retardation test, which shows that CI was developed. Finally, those subjects that received five A+ and 11 AX**−** trials per session (Experiment 3) did not show an increase in responding to X at any moment of the experiment, proving that SOC was not developed, but they did show a retarded acquisition of conditioning to X in the retardation test, congruent with the development of CI.

Taken together, these results indicate that both the number of A+ and AX**−** trials and the progression of the sessions are important variables that determine if SOC or CI would occur. These variables also seem to interact with each other. SOC appeared with many A+ and few AX**−** trials as well as with an equal number of A+ and AX**−** trials (14–2 and 8–8, respectively), but as the sessions progressed, SOC was no longer evident. CI is demonstrated at the end of training with an equal number of A+ and AX**−** trials, with few A+ and many AX**−** trials (8–8 and 5–11, respectively). It is worth noting that the results of the present study are consistent with the previous ones in which the number of AX**−** trials per session was manipulated (Yin et al., 1994; Stout et al., 2004), with the novelty that, in this study, the total number of trials per session and the ITI was held constant. However, as both A+ and AX**−** trials were varied, it is not clear if the results found were due to the number of A+ trials, the number of AX**−** trials, or the conjoint effect of both trial numbers. Further investigation is needed to address this question.

Furthermore, group 8-8Exp in Experiment 2 replicates the findings reported by Rescorla (1972, 1973) and by Holland and Rescorla (1975), as SOC is shown at the beginning of training and CI is observed at the end of training. CI was assessed with different tests. Whereas previous studies employed a summation test to assess the inhibitory properties of the additional cue X, in the present study a retardation test was used. It has been argued that using both summation and retardation tests, the so-called two-test strategy, is the best way to test the inhibitory properties of a stimulus as it allows one to rule out alternative explanations based on attentional shifts (Rescorla, 1969). Reduced attention to a stimulus can account for the retardation effect but will not affect responding to a transfer excitor, i.e., there will be no reduction in responding in the summation test. Conversely, increased attention to a stimulus would decrease responding to a transfer excitor in a summation test but would not produce a retardation effect, so if a stimulus passes both summation and retardation tests, it cannot be due to an attentional shift. However, some authors have claimed that both tests might not be sufficient nor necessary to assess the inhibitory properties of a stimulus (Williams et al., 1992). In fact, according to Papini and Bitterman (1993), in the A+/AX**−** design, a retardation test would be sufficient as long as the experiment includes a control group in which the putative inhibitory stimulus receives a treatment that is assumed to be less inhibitory or not inhibitory at all compared to the treatment received by the experimental group, as is the case of the present experiments. According to Papini and Bitterman (1993), as in the present study a control group trained with a BX compound was included, a retardation test could be sufficient, given that attention cannot readily be assumed to be less in the experimental than in the control group.

**FIGURE 7:**
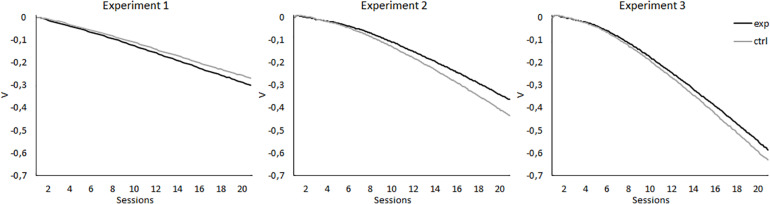
Simulations of associative properties acquired by X according to Wagner’s Sometimes Opponent Processes (SOP) model, performed using the SOP model simulator (Byers et al., 2017), for the 20 sessions of the experimental designs reported. The left panel displays the simulation for Experiment 1, in which the experimental group (black line) was trained with 14 A+, two AX**−**, 12 F**−**, and two X**−** trials per session, and the control group (gray line) was trained with 14 A+, two BX**−**, 12 F**−**, and two X**−** trials per session. The central panel displays the simulations for Experiment 2, in which the experimental group (black line) was trained with eight A+, eight AX**−**, six F**−**, six F+, and two X**−** trials per session, and the control group (gray line) was trained with eight A+, eight BX**−**, six F**−**, six F+, and two X**−** trials per session. The right panel displays the simulations for Experiment 3, in which the experimental group (black line) was trained with five A+, 11 AX**−**, three F**−**, nine F+, and two X**−** trials per session, and the control group (gray line) was trained with five A+, 11 BX**−**, three F**−**, nine F+, and two X**−** trials per session.

As noted earlier, these results are challenging for most theories of associative learning, as most of these models simply cannot predict the existence of SOC (e.g., Rescorla and Wagner, 1972; Mackintosh, 1975; Pearce and Hall, 1980; Pearce, 1987). However, Stout et al. (2004) noticed that their results might be explained by the models proposed by Wagner (1981), by Sutton and Barto (1981), and by McLaren and Mackintosh (2000). These models explain SOC as an associative chain involving an association between X and A and an association between A and the US, so X indirectly activates the representation of the reinforcer. They are also able to explain CI, given that, with extended training, X is associated with the absence of the reinforcer that was expected due to the presence of A. However, this explanation of SOC requires the treatment to be performed in two phases, that is, A+ should be first conditioned to the asymptotic level in a phase prior to the presentation of the AX**−** compound. It is only under these circumstances that A can function as a reinforcer for X. To illustrate this, simulations of Wagner’s Sometimes Opponent Processes (SOP) model for the present experiments were performed using the SOP model simulator (Byers et al., 2017). As can be seen in the left panel of Figure 7, in Experiment 1, for both the group that was trained with 14 A+ and two AX**−** trials per session and the group that was trained with 14 A+ and two BX**−** trials per session, the model predicts the development of an inhibitory link between X and the US, not predicting that X would gain excitatory properties at any point of the experiment. It is worth noting that, although it does not predict SOC, the predictions are consistent with the results on the retardation test, where both groups showed similar levels of responding. The simulations for Experiment 2 are displayed in the central panel of Figure 7. For both the group that was trained with eight A+ and eight AX**−** per session and the group that was trained with eight A+ and eight BX**−** per session, the model predicts that X would develop inhibitory properties, with the strength of this inhibition being stronger in the control group. Thus, the results of the simulations are not consistent with the results obtained in Experiment 2, as they predict neither the development of SOC during the first sessions nor the retardation that X shows in the experimental group in the retardation test. For Experiment 3, the model predicts an inhibitory relationship between X and the US for both groups, with the inhibition being stronger in the control group, especially in the last sessions. The results of the simulations are consistent with the absence of excitatory properties of X in the first sessions of Experiment 3, but not with the results of the retardation test, given that in the experiment it was found that X had acquired inhibitory properties in the 5-11Exp group compared with the 5-11Ctrl group. In conclusion, for the present experiments, this model cannot predict SOC through an associative chain that involves the association between X and A and the association of A with the US. It does predict the acquired inhibitory properties for X. However, it does not predict the results of the retardation tests, as according to the model, X would acquire similar or stronger inhibitory properties for the control groups than for the experimental ones.

Another significant exception are the models that distinguish between acquisition and performance (see Miller, 2006, for a review). The models described previously share the assumption that the response to a stimulus depends only on the associative status of that stimulus and that cue competition occurs in acquisition. In performance-focused models, such as the comparator hypothesis proposed by Miller and Matzel (1988), associations are acquired in a non-competitive fashion, in such a way that all associations are excitatory, and inhibition is a result of the interaction between them, so inhibition is due to a process of comparison between stimuli at the moment of responding, in such a way that responding to a stimulus depends not only on its association with the reinforcer but also on the association with the reinforcer that has been acquired by other stimuli. In our experiments, CI would be the result of this comparison process, as the association between X and the reinforcer is 0, given that they are never presented together, and the comparison term value is high, as it depends on the association between X and A, and the association between A and the reinforcer. SOC would be predicted by the presence of a switching operator in the response rule that makes the result of the comparison excitatory in the first sessions and that, with the repeated presentation of the stimulus X, switches so that the net result of the comparison becomes inhibitory (Stout and Miller, 2007). It is worth noting that Pineño (2007) proposed a similar response rule but that can be applied in conjunction with acquisition rules from competitive acquisition models. According to this rule, competition occurs during acquisition, whereas facilitation occurs during performance, as a result of summing the associative strength of the stimulus X and the associative strength of the stimuli associated with it, weighted by the strength of the within-stimuli association and the novelty of the stimulus X. The transition from facilitation to competition is due to the decreased novelty of the stimulus X as training progresses. Although the acquisition mechanism is different in these two proposals and the comparison process in responding is slightly different, both can account for the present results.

## Conclusion

To sum up, the present set of experiments provide a demonstration of the modulatory effect of the number of trials per session and the number of sessions on associative learning phenomena, adding evidence to the available literature that demonstrates that cue interactions can be facilitative and competitive (Urcelay, 2017). The results presented here are problematic for most learning theories, being more easily explained by theories that distinguish between what is learned and what is overtly displayed through behavior.

## Data Availability Statement

The raw data supporting the conclusions of this article will be made available by the authors, without undue reservation.

## Ethics Statement

The animal study was reviewed and approved by the Animal Welfare Committee of the University of Oviedo.

## Author Contributions

CM-D, JM-M, and IL contributed to the study design and final revision, and approved the final version of the manuscript. CM-D implemented the study, analyzed the results, and wrote the manuscript. JM-M contributed to the implementation of the study. JM-M and IL revised the manuscript. All the authors contributed to the article and approved the submitted version.

## Conflict of Interest

The authors declare that the research was conducted in the absence of any commercial or financial relationships that could be construed as a potential conflict of interest.
